# Can remote STI/HIV testing and *e*Clinical Care be compatible with robust public health surveillance?

**DOI:** 10.1145/2750511.2750517

**Published:** 2015-05-18

**Authors:** Emma Harding-Esch, Anthony Nardone, Jo Gibbs, Lorna Sutcliffe, Pam Sonnenberg, Claudia Estcourt, Gwenda Hughes, Hamish Mohammed, Noel Gill, S Tariq Sadiq, Catherine Lowndes

**Affiliations:** HIV/STI Department Public Health England London. +44 (0)20 8327 6520 emma.harding-esch@phe.gov.uk; HIV/STI Department Public Health England London. +44 (0)20 8327 6948 anthony.nardone@phe.gov.uk; Queen Mary University of London, St Bartholo mew’s Hospital; Queen Mary University of London, St Bartholomew’s Hospital; UCL Centre for Sexual Health & HIV Research, University College London; Queen Mary University of London, Barts Sexual Health Centre, St Bartholomew’s Hospital; HIV/STI Department, Public Health England, London; HIV/STI Department, Public Health England, London; HIV/STI Department, Public Health England, London; Institute for Infection and Immunity, St George’s University of London; HIV/STI Department Public Health England London. +44 (0)20 8327 6711 catherine.lowndes@phe.gov.uk

**Keywords:** Public health, surveillance, HIV, STI, Sexually transmitted infection, eClinical care, patient pathways, self-testing

## Abstract

In this paper we outline the current data capture systems for human immunodeficiency virus (HIV) and sexually transmitted infection (STI) surveillance used by Public Health England (PHE), and how these will be affected by the introduction of novel testing platforms and changing patient pathways. We outline the Chlamydia Online Clinical Care Pathway (COCCP), developed as part of the Electronic Self-Testing for Sexually Transmitted Infections (*e*STI^2^) Consortium, which ensures that surveillance data continue to be routinely collected and transmitted to PHE. We conclude that both novel diagnostic testing platforms and established data capture systems must be adaptable to ensure continued robust public health surveillance.

## 1. INTRODUCTION

Public Health England (PHE) collects comprehensive electronic surveillance data from a variety of sources. In the human immunodeficiency virus (HIV) and sexually transmitted infection (STI) Department, these include data on numbers of STI tests, diagnoses, and associated epidemiological information from sexual health services (GUMCAD), data on all chlamydia tests and outcomes from laboratories (CTAD), data on antimicrobial resistance patterns for gonorrhea from linked microbiological and clinical data (GRASP), data on HIV and AIDS reporting covering all service providers of English HIV outpatient services (HARS), and data on the number of patients who recently acquired HIV infection at the time of diagnosis (RITA). Consequently, England has timely, comprehensive and sophisticated HIV and STI surveillance systems, which compare favorably with those in other western industrialized countries.

Although internet requesting of STI sampling kits for home use is widely available in the UK, users return their samples to the laboratory for testing, and ongoing management follows traditional care pathways. Therefore, there is minimal impact on data acquisition for surveillance purposes. Similarly for HIV, pilots of an HIV self-sampling service accessed through the internet have proved very successful in the UK and this is now already considered part of standard of care.

New diagnostic test platforms, such as rapid tests and Point-of-Care (PoC) technologies, have the potential to be used in novel settings such as people’s homes (home or self-sampling/self-testing). An HIV self-test was licensed in early 2014, and although there is as yet no CE-marked kit available on the market, there are both anticipated impacts on surveillance because of the difficulties in monitoring those who are being tested, as well as the challenge of ensuring positive results are linked into care services for confirmation, treatment and care. Data from England’s National Chlamydia Screening Programme (NCSP) show that 7% of chlamydia testing is internet-based, with high positivity of 11%, demonstrating the importance of being able to capture surveillance-related data from those being tested with new diagnostic technologies, for the meaningful assessment of testing patterns and trends in STI rates.

## 2. CHANGING PATIENT PATHWAYS

**Figure 1:**
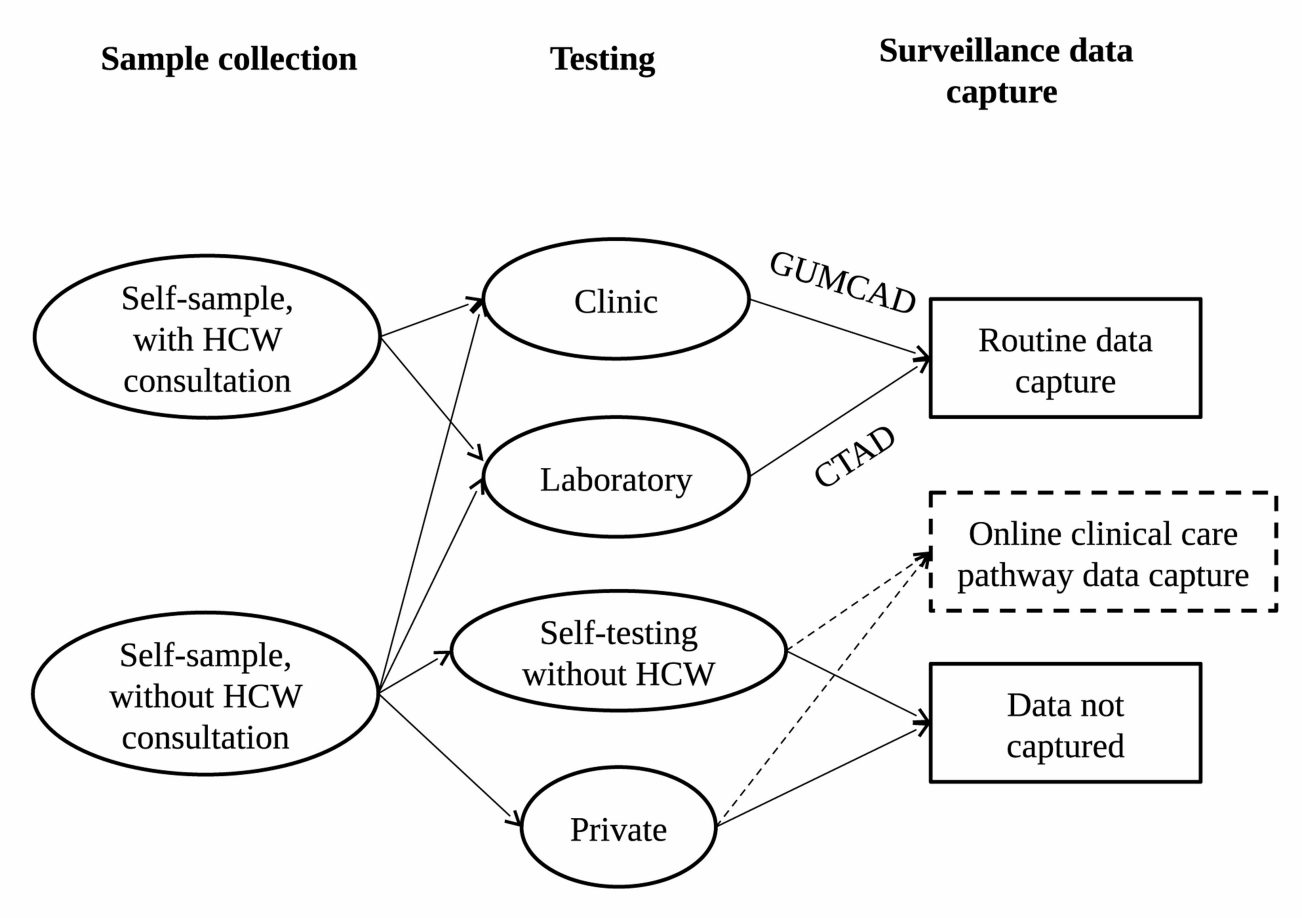
Patient pathways involving established and novel sample collection, testing and data capture mechanisms.

Surveillance systems and their data sources will therefore need to adapt and be ready as these tests become cheaper, more accurate, and more widely available. This is particularly true if these tests become de-coupled from traditional care pathways. For example, the electronic self-testing for sexually transmitted infections (*e*STI^2^) consortium aims to reduce the high impact of infectious diseases by linking the capacity to develop and implement simple to use, rapid, accurate tests for multiple infections which are affordable, reliable and can be mobile-phone networked, to on-line clinical care pathways (http://www.esti2.org.uk/). Figure 1 depicts the current general patient pathways for sample collection, testing, and data transmission for surveillance purposes of STIs in England.

Self-sampling can be done either as part of a health care worker (HCW) consultation, or without HCW involvement. Samples from both these methods can be sent for established testing methods, at the clinic or direct to laboratories, where data are routinely transmitted to PHE (STI clinics to GUMCAD, laboratories to CTAD). However, a self-collected sample without a HCW can also be tested using novel rapid or PoC tests. These could become available through routine services, or they could be privately bought tests. Private market testing and diagnosis data are already not captured by existing surveillance systems, but the advent of novel sampling and testing methods could increase the size of the private market. Rapid and PoC testing, and increase in private market tests, will result in loss of data capture by PHE, unless they can be coupled with a new data capture system, such as an online clinical care pathway.

As part of *e*STI^2^, the Chlamydia Online Clinical Care Pathway (COCCP) has been developed. This *e*Clinical care pathway focuses on the UK’s most commonly diagnosed STI (*Chlamydia trachomatis*). The pathway enables people with genital chlamydia to receive their test result online, obtain information about the infection, complete a clinical consultation, and progress to receive a remote prescription of antibiotic treatment, within “*e*Sexual Health clinics”, embedded within England’s National Health Service (NHS) sexual health services. An important part of the development of the *e*Clinical Care Pathway Framework was ensuring that surveillance data continued to be routinely collected and transmitted to PHE. The COCCP was specifically designed to capture all data items routinely collected in traditional consultations for public health surveillance purposes and for the data to be captured through established mechanisms (GUMCAD for STI clinic data, and CTAD for laboratories). The system was also designed to be interoperable so that in the future, surveillance data could be fed directly from the COCCP to PHE and that data could be captured through information input via the internet, or into a mobile phone or other hand-held device by the PoC test user. However, the success of such pathways will be dependent on the willingness of users to provide their data, particularly test-negative patients who would have no contact with services once they received their test result, and for whom surveillance data would need to be collected prior to receiving their test result.

Although there may be a risk that these new care pathways would cater for the “worried-well”, they may also increase testing in high risk populations by removing barriers to testing in current traditional and outreach settings. The advent of new technologies may also make data harder to interpret, as trends observed may result from changing population testing profiles as opposed to intrinsic changes in infection rates. Interpretation is further complicated by the need for identification and removal of duplicates (individuals tested more than once, for example, in community and clinical settings) once the data have been transmitted to PHE. Although deduplication is already an issue in existing surveillance systems, home sampling and testing will likely add to this problem as individuals test at home and then attend a more traditional setting for confirmatory testing. Identifying when confirmatory testing is taking place would have the additional advantage of verifying the initial PoC test result. Further, having a CE-mark does not guarantee that the test has high sensitivity or specificity. If there are no robust controls of tests available to buy for home- or self-testing, or pathways to confirm test result, patients may receive inaccurate diagnoses and management.

## 3. CONCLUSIONS

In conclusion, a critical concern for any established surveillance system currently dependent on data obtained from public sources, is the loss of data as individuals no longer engage with established services and are instead tested and managed remotely. If online self-testing, such as that described here, was commissioned by a public body, such as the National Health Service, public health monitoring would need to be part of the pathway. Mechanisms of data capture for surveillance purposes could also be incorporated into private market tests. There is flexibility in the existing surveillance structure to adapt to, and accommodate, novel tests and patient management systems. Engaging with those responsible for established STI/HIV surveillance would need to take place early on in the development of remote care pathways.

